# Design and Modeling of Polysilicon Electrothermal Actuators for a MEMS Mirror with Low Power Consumption

**DOI:** 10.3390/mi8070203

**Published:** 2017-06-25

**Authors:** Miguel Lara-Castro, Adrian Herrera-Amaya, Marco A. Escarola-Rosas, Moisés Vázquez-Toledo, Francisco López-Huerta, Luz A. Aguilera-Cortés, Agustín L. Herrera-May

**Affiliations:** 1 Micro and Nanotechnology Research Center, Universidad Veracruzana, Calzada Ruiz Cortines 455, Boca del Río, VER 94294, Mexico; septmig@gmail.com (M.L.-C.); maerescarola@gmail.com (M.A.E.-R.); leherrera@uv.mx (A.L.H.-M.); 2 Depto, Ingeniería Mecánica, Campus Irapuato-Salamanca, Universidad de Guanajuato/Carretera Salamanca-Valle de Santiago Km. 3.5 + 1.8 km, Salamanca, GTO 36885, Mexico; herreraugto@gmail.com (A.H.-A.); aguilera@ugto.mx (L.A.A.-C.); 3 Sistemas Automatizados, Centro de Ingeniería y Desarrollo Industrial/Av. Pie de la Cuesta No. 702, Desarrollo San Pablo, Querétaro 76125 México; moises.vazquez@cidesi.edu.mx; 4 Engineering Faculty, Universidad Veracruzana, Calzada Ruiz Cortines 455, Boca del Río, Veracruz 94294, Mexico

**Keywords:** electrothermal actuators, endoscopic optical-coherence tomography, microelectromechanical systems (MEMS) mirror, polysilicon, SUMMiT V

## Abstract

Endoscopic optical-coherence tomography (OCT) systems require low cost mirrors with small footprint size, out-of-plane deflections and low bias voltage. These requirements can be achieved with electrothermal actuators based on microelectromechanical systems (MEMS). We present the design and modeling of polysilicon electrothermal actuators for a MEMS mirror (100 μm × 100 μm × 2.25 μm). These actuators are composed by two beam types (2.25 μm thickness) with different cross-section area, which are separated by 2 μm gap. The mirror and actuators are designed through the Sandia Ultra-planar Multi-level MEMS Technology V (SUMMiT V^®^) process, obtaining a small footprint size (1028 μm × 1028 µm) for actuators of 550 µm length. The actuators have out-of-plane displacements caused by low dc voltages and without use material layers with distinct thermal expansion coefficients. The temperature behavior along the actuators is calculated through analytical models that include terms of heat energy generation, heat conduction and heat energy loss. The force method is used to predict the maximum out-of-plane displacements in the actuator tip as function of supplied voltage. Both analytical models, under steady-state conditions, employ the polysilicon resistivity as function of the temperature. The electrothermal-and structural behavior of the actuators is studied considering different beams dimensions (length and width) and dc bias voltages from 0.5 to 2.5 V. For 2.5 V, the actuator of 550 µm length reaches a maximum temperature, displacement and electrical power of 115 °C, 10.3 µm and 6.3 mW, respectively. The designed actuation mechanism can be useful for MEMS mirrors of different sizes with potential application in endoscopic OCT systems that require low power consumption.

## 1. Introduction

Microelectromechanical systems (MEMS) have allowed the develop of devices with advantages such as low cost, small size, high reliability, fast response and easy integration with electronic circuits [1,2,3]. Among these devices, MEMS mirrors have potential applications such as projection displays [4], tunable optical filter [5], tunable laser [6], Fourier transform spectrometer system [7], confocal scanning microendoscope [8], optical bio-imaging [9] and optical coherence tomography [10]. For 3D endoscopic optical-coherence tomography (OCT) systems are necessary low cost MEMS mirrors composed by compact structures that have large out-of-plane deflections, minimum bias voltage and orthogonal scanning capacity [11,12]. These systems are minimally invasive and can have high resolution and reliability [12]. For this, the mirrors need high precision actuators that allow the variation of their tilting angles with low power consumption [13]. To adjust and control the mirror motion can use different actuators types, including the electromagnetic [14,15], electrostatic [16], electrothermal [17,18] or piezoelectric [19,20] actuators. 

Mirrors with electrostatic actuators have a fast speed, a small mechanical scanning range at non-resonance (generally 2°–3°) and a large actuator footprint, which can be increased at resonance [21,22]. This actuation mechanism requires complex fabrication and high drive voltages about 100 V [23], which constraints its application in endoscopic OCT systems. Other actuators are the electromagnetics that generate large displacements with small driving voltage and have fast response time as well as high resonance frequency [24,25,26]. Although electromagnetic mirrors register problems with electromagnetic interference (EMI) and need precise assembly techniques of magnetic materials and metallic coils, limiting they use in endoscopic imaging [26]. On the other hand, piezoelectric actuators offer a large motion range combined with high speed and low electric energy [27]. Nevertheless, there are several challenges of the MEMS mirrors to develop endoscopic imaging such as charge leakage, coupling nonuniformity and hysteresis [28]. Other option is a MEMS mirror with an electrothermal actuation mechanism, which has large deflections caused by low bias voltage and does not present EMI and electrostatic discharging problems [28,29,30,31,32]. However, these mirrors require to decrease their footprint size, operation temperature and bias voltage as well as simplify their mechanical structure and performance. To overcome several of these challenges, we propone the design of polysilicon electrothermal actuators for MEMS mirrors based on the Sandia Ultra-planar Multi-level MEMS Technology V (SUMMiT V^®^) process from Sandia National Laboratories. This electrothermal actuation mechanism has a simple structural configuration composed by an array of four polysilicon actuators, which can achieve out-of-plane displacements with low dc voltages. These actuators do not require materials with different thermal expansion coefficients due to that employ polysilicon layers with distinct wide, which are separated by 2 μm gap. This device has a small footprint size (1028 μm × 1028 μm), compact structure and simple performance with reduced temperatures. The proposed design includes the modeling of temperature behavior and maximum displacements of the actuators under steady-state conditions. Our actuation mechanism can be used for the rotation of MEMS mirrors of different sizes. The rotation orientation of the mirror can be adjusted through the selective biasing of the four actuators. Thus, the proposed design could be considered for potential applications in endoscopic OCT systems.

This paper is organized as follows. Section 2 contains the design and modeling of the proposed actuation mechanism, which includes its electrothermal and structural behavior. Section 3 shows the results and discussions of temperature and out-of-plane displacements of the actuators using analytical models. Finally, the paper ends with the conclusion and future researches.

## 2. Design and Modeling

This section presents the design and modeling of the electrothermal actuators for a MEMS mirror. It considers the temperature distribution and out-of-plane displacements of the actuators generated by different dc biasing voltages under steady-state conditions.

### 2.1. Structural Configuration

**Figure 1:**
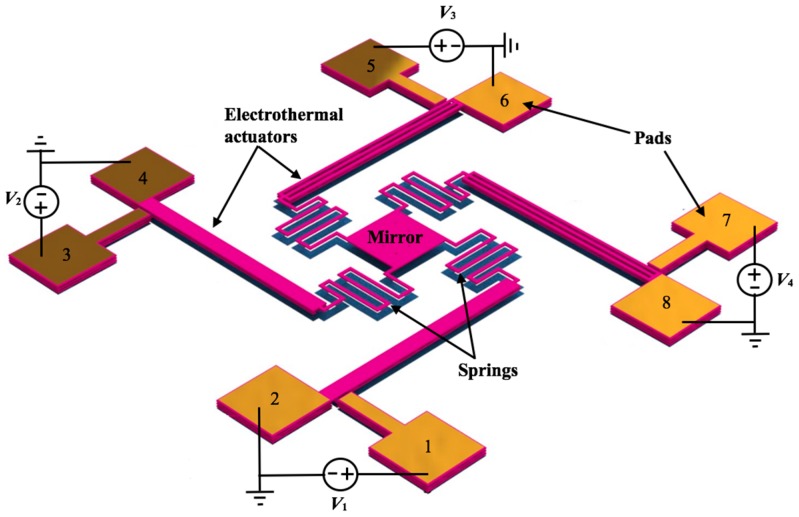
Design of an electrothermal actuation mechanism for the rotation of a microelectromechanical systems (MEMS) mirror.

**Figure 2:**
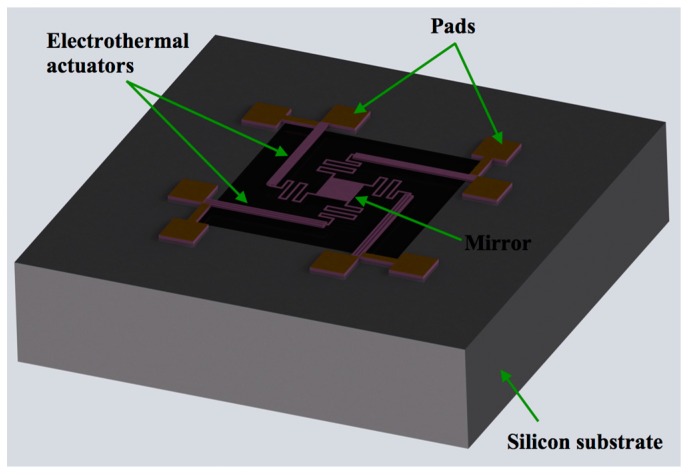
View of the MEMS mirror design in a silicon die.

**Figure 3:**
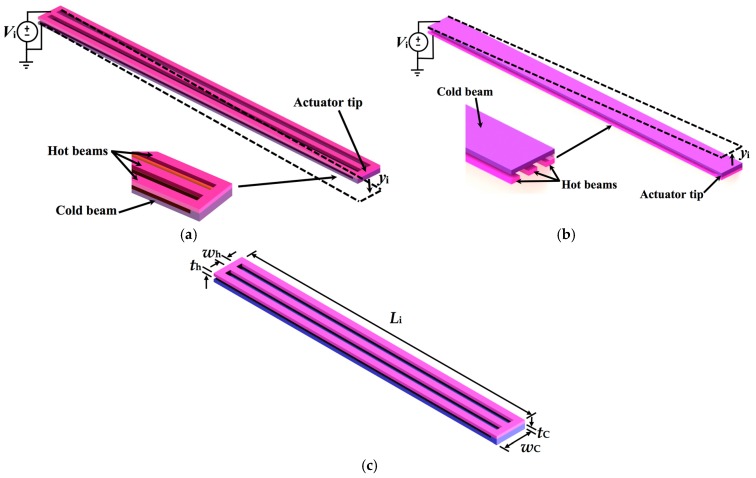
View of out-of-plane displacements, *y_i_*, with directions (**a**) downward and (**b**) upward of two electrothermal actuators with inverted structural layers due to Joule effect; (**c**) geometrical parameters of the hot and cold beams of an electrothermal actuator.

Figure 1 shows the design of a MEMS mirror with an array of four polysilicon electrothermal actuators and springs, which are based on the SUMMiT V process [33]. The surface of the silicon substrate below of the actuators and mirror must be etched to allow the free motion of the actuators and mirror, as shown in Figure 2. Each actuator has two polysilicon structural layers (i.e., poly3 and poly4 of the SUMMiT V process) of 2.25 μm thickness with different cross-section area, separated by 2 μm gap. Thus, the electrical resistances of these layers are not equal, which allow a temperature change along the actuator when an electrical current is applied. It generates out-of-plate displacements of the actuator due to Joule effect, whose amplitudes can be controlled varying the current values. Thus, this actuator does not need materials layers with different thermal expansion coefficients that simplify its fabrication process. This design includes actuators with inverted structural layers to achieve out-of-plane motions with opposite directions, as shown in Figure 3a,b. Thereby, the mirror is connected to two pair actuators with inverted layers that can have displacements in opposite directions, increasing the tilting angle of the mirror. In addition, four polysilicon springs (508 μm length, 5 μm width and 2.25 μm thickness) with low stiffness are employed to connect the actuators with the mirror. Due to the small cross-section area and large length of each spring, the four springs have high electrical resistance that constraint the current flow through them. In this work, the effect of the thermal energy through the springs and mirror is not considered.

In the design stage, the temperature and out-of-plane displacements of the actuators considering different dimensions of length (*L_i_*) and width (*ω_h_* and *ω_c_*) of the upper (hot) and bottom (cold) beams are studied. The first structural layer is formed by a polysilicon beam (*ω_c_*) and the second layer is composed by three polysilicon beams of width *ω_h_* each one, in which *ω_h_* << *ω_c_*. Figure 3a,b depicts views of the hot and cold beams in two electrothermal actuators with deflections in opposite directions. In addition, the mirror and springs are designed using the poly4 layer of SUMMiT V process. In this fabrication process, on the mirror surface can be deposited an aluminum layer (96 μm × 96 μm × 0.7 μm). The springs have a connection with low stiffness between the actuators and mirror, which lets higher mirror tilting. 

The operating principle of the electrothermal actuator with bending motion is caused by the asymmetrical thermal expansion of the two structural layers with different cross section area and electrical resistance. The resistance of the narrower layer is higher than that of the wider layer. If a dc bias voltage is applied at the end of the two layers (see Figure 3a,b) then a current flows through them, generating an increase of temperature in both layers. Due to the difference in the electrical resistance of the two layers, the temperature and dissipated energy in the narrower layer (high electrical resistance) is larger than the wider layer (low electrical resistance). This allows more thermal deformation of the narrower layer, which forces the actuator tip to an out-of-plane motion towards the wider layer. Therefore, the difference of the thermal deformation between the two actuator layers generates an out-of-plane motion. Figure 3c depicts the main geometrical parameters of an electrothermal actuator. In this work, we consider actuators with three different lengths (350 μm, 450 μm and 550 μm), constant thickness (*t_h_* = *t_c_* = 2.25 μm) and variable width (i.e., *w_h_* of 2 μm to 5 μm and *w_c_* of 20 μm to 30 μm). 

### 2.2. Electrical Model of Electrothermal Actuators

**Figure 4:**
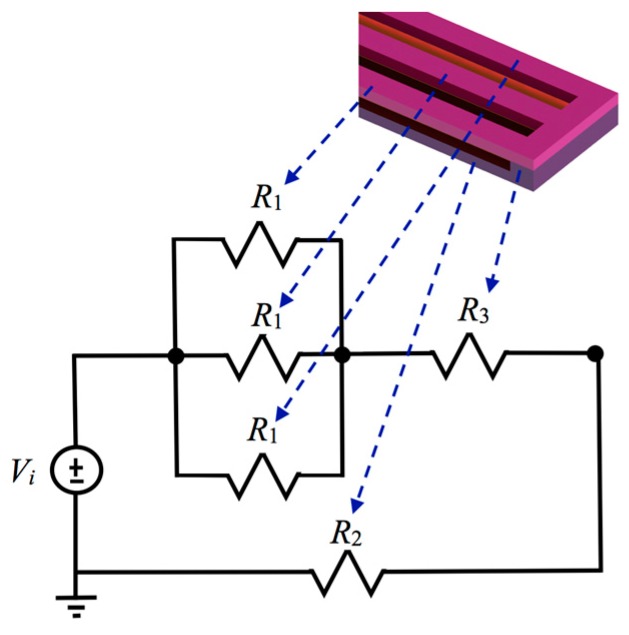
Schematic of equivalent electrical circuit of an electrothermal actuator.

An equivalent electric circuit of the electrothermal actuator is developed to predict the voltage drop along its hot and cold beams, as shown in Figure 4. For this case, *R*_1_, *R*_2_ and *R*_3_ are the electrical resistance values obtained for each hot beam (*ω_h_*), cold beam (*ω_c_*) and connection between both beams, respectively. These resistances are calculated including the dimensions of the beams and the resistivity of the polysilicon layers. For instance, Table 1 shows the values of the electrical resistances for an electrothermal actuator with the following dimensions: *L_h_* = *L_c_* = 450 μm, *ω_h_* = 5 μm, *ω_c_* = 30 μm and *t_h_* = *t_c_* = 2.25 μm. 

### 2.3. Analytical Modeling of the Electrothermal and Structural Behavior

**Figure 5:**
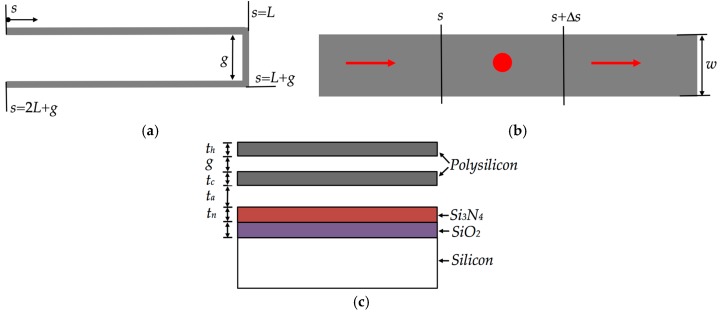
(**a**) Schematic of the one-dimensional model for an electrothermal actuator; (**b**) its differential element; and (**c**) cross-section of the different layers for the thermal analysis.

The electrothermal behavior of a polysilicon beam with length larger than its thickness and width can be simplified using an analysis in one dimension [31]. The electrothermal actuator (see Figure 3a) can be decomposed into three line-shape beams connected in series. For this, the first line-shape beam is obtained combining the three upper beams (hot beams) in a wider beam. Thus, the first line-shape beam has an equivalent electrical resistance equal to a third of the resistance of an upper beam. The second line-shape beam is formed by the connection between the upper and bottom beams, which has a 2.5 μm gap. In addition, the bottom (cold) beam forms the third line-shape beam. For this case, we assumed that the length of the upper (hot) beam (*L_h_*) is equal to the length of the bottom beam (*L_c_*): *L_h_* = *L_c_* = *L*. Figure 5 shows a differential element for the thermal analysis of the actuator.

In Figure 5b, heat flow equation is obtained by examining a differential element of polysilicon beam of width *w*, thickness *t* and length *Δs*. Assuming steady-state conditions, resistive heating power in the differential element is equal to heat conduction out of the element. Therefore, the energy balance of the differential element of the beam with heat losses can be expressed as [31]:

(1) $$-k_{p}wt{\left[\frac{dT}{ds}\right]}_{s}+J^{2}\rho wt\Delta s-Q\Delta sw\frac{T-T_{0}}{R_{t}}=-k_{p}wt{\left[\frac{dT}{ds}\right]}_{s+\Delta s}$$

where *J* is the current density, *k_p_* is the thermal conductivity and *ρ* is the resistivity of the polysilicon, *T* is the operation temperature, *T*_0_ is the substrate temperature, *Q* is the shape factor that includes the impact of the element shape on heat conduction to the substrate and *R_t_* is the thermal resistance generated by the substrate and actuator that are considered wide enough [31]:

(2) $$R_{t}=\frac{t_{a}}{k_{a}}+\frac{t_{n}}{k_{n}}+\frac{t_{s}}{k_{s}}$$

where *t_a_* is the distance between both the bottom beam of the actuator and Si_3_N_4_ surface, *t_n_* is the thickness of the Si_3_N_4_ film, *t_s_* is the thickness of the SiO_2_ film and *k_a_*, *k_n_* and *k_s_* are the thermal conductivity of air, Si_3_N_4_ and SiO_2_ films, respectively.

The shape factor *Q* for the heat conduction is given by [34]:

(3) $$Q=\frac{t}{w}\left(\frac{2t_{a}}{t}+1\right)+1$$

To apply Equation (3) in the electrothermal actuator, we approximated *t* = *t_h_* = *t_c_* and *w* = *w_c_*.

The resistivity of polysilicon, *ρ*(*T*), depends of the temperature and its value is determined by:

(4) $$\rho (T)=\rho_{0}\left[1+\xi \left(T-T_{0}\right)\right]$$

where $\rho_{0}$ is the initial resistivity at the substrate temperature and *ξ* is the linear temperature coefficient. 

Considering the limit as Δs→0 for Equation (1), the following second-order differential equation is obtained:

(5) $$k_{p}\frac{d^{2}T}{ds^{2}}+J^{2}\rho =\frac{Q}{t}\frac{\left(T-T_{0}\right)}{R_{t}}$$

The first term on the left of Equation (5) indicates the net rate of heat conduction into the element per unit volume. The rate of heat energy generation inside the element per unit volume is represented by the second term on the left. Finally, the rate of heat energy loss of the element per unit volume is considered in the term of the right side. Substituting Equation (4) into Equation (5), we obtain:

(6) $$\frac{d^{2}T}{ds^{2}}-m^{2}T=-m^{2}T_{0}-\frac{J^{2}\rho_{0}}{k_{p}}$$

with

(7) $$m^{2}=\frac{Q}{k_{p}R_{t}t}-\frac{J^{2}\rho_{0}\xi }{k_{p}}$$

Solving Equation (6) and applying the solution to the upper (hot) and bottom (cold) beams, we get the following temperature distribution:

(8) $$T_{h}(s)=C_{1}e^{m_{h}s}+C_{2}e^{-m_{h}s}+T_{o}+\frac{J_{h}^{2}\rho_{0}}{k_{p}m_{h}^{2}}$$

(9) $$T_{c}(s)=C_{3}e^{m_{c}s}+C_{4}e^{-m_{c}s}+T_{o}+\frac{J_{c}^{2}\rho_{0}}{k_{p}m_{c}^{2}}$$

with

(10) $$m_{h}^{2}=\frac{Q}{k_{p}R_{t}t}-\frac{J_{h}^{2}\rho_{0}\xi }{k_{p}}$$

(11) $$m_{c}^{2}=\frac{Q}{k_{p}R_{t}t}-\frac{J_{c}^{2}\rho_{0}\xi }{k_{p}}$$

where *T_h_*(*s*) and *T_c_*(*s*) are the temperature distribution along the upper (hot) and bottom (cold) beams, respectively, and *J_h_* and *J_c_* are the current density through the upper and bottom beams, respectively. 

To determine the constants *C_i_*, we assume a temperature on the anchor pads equal to the substrate temperature (i.e., *T_h_*(0) = *T*_0_ and *T_c_*(2*L* + *g*) = *T*_0_), a continuity of both temperature (i.e., $T_{h}\left(L\right)=T_{c}\left(L\right)$) and rate of heat conduction (i.e., 3*w_h_dT_h_*(*L*)/*ds* = *w_c_dT_c_*(*L*)/*ds*) across the join point of the upper and bottom beams. By assuming these boundary conditions, the following matrix equation is determined as:

(12) $$\left[\begin{array}{cccc} 1 & 1 & 0 & 0\\ e^{m_{h}L} & e^{-m_{h}L} & -e^{m_{c}L} & -e^{-m_{c}L}\\ 3\omega_{h}m_{h}e^{m_{h}L} & -3\omega_{h}m_{h}e^{-m_{h}L} & -\omega_{c}m_{c}e^{m_{c}L} & \omega_{c}m_{c}e^{-m_{c}L}\\ 0 & 0 & e^{m_{c}(2L+g)} & e^{-m_{c}(2L+g)} \end{array}\right]\left[\begin{array}{c} C_{1}\\ C_{2}\\ C_{3}\\ C_{4} \end{array}\right]=\left[\begin{array}{c} -\frac{J_{h}^{2}\rho_{0}}{k_{p}m_{h}^{2}}\\ \frac{J_{c}^{2}\rho_{0}}{k_{p}m_{c}^{2}}-\frac{J_{h}^{2}\rho_{0}}{k_{p}m_{h}^{2}}\\ 0\\ -\frac{J_{c}^{2}\rho_{0}}{k_{p}m_{c}^{2}} \end{array}\right]$$

The coefficients *C_i_* of Equation (12) are determined using operations on matrices. Next, these coefficients are employed into Equations (8) and (9) to calculate the temperature increase along the upper and bottom beams due to bias voltages. These coefficients are calculated as:

(13) $$C_{1}=\frac{A\left(3+d\right)-e^{m_{h}L}\left[Bd\left(1+e^{2m_{c}(L+g)}\right)+2Dde^{m_{c}(L+g)}\right]-A\left(3-d\right)e^{2m_{c}(L+g)}}{\left(d-3\right)e^{2m_{c}(L+g)}-\left(d+3\right)e^{2(m_{c}(L+g)+m_{h}L)}+e^{m_{h}L}\left(3-d\right)\left(e^{m_{h}L}+e^{-m_{h}L}\right)}$$

(14) $$C_{2}=\frac{\left(Bd+A\left(3-d\right)e^{m_{h}L}+\left(d\left(B+2D\right)-A\left(3+d\right)e^{m_{h}L}\right)e^{2m_{c}(L+g)}\right)e^{m_{h}L}}{\left(d-3-\left(d+3\right)e^{2m_{h}L}\right)e^{2m_{c}(L+g)}+2\left(3\cosh(m_{h}L)-d\sinh(m_{h}L)\right)e^{m_{h}L}}$$

(15) $$C_{3}=\frac{D\left(2\left(9+d^{2}\right)+\left(9-d^{2}\right)\left(e^{-2m_{h}L}+e^{2m_{h}L}\right)\right)+18Be^{-m_{c}(L+g)}+F+24A\left(G+H\right)}{4\left(G+H\right)\left(\left(3-d\right)e^{(m_{c}+m_{h})L}+\left(3+d\right)e^{(m_{c}-m_{h})L}\right)}$$

(16) $$C_{4}=\frac{\left(-6Ae^{m_{c}(2L+g)}+D\left(\left(3+d\right)e^{-m_{h}L}+\left(3-d\right)e^{m_{h}L}\right)e^{m_{c}L}+3Be^{m_{c}(2L+g)}\left(e^{m_{h}L}+e^{-m_{h}L}\right)\right)e^{2m_{c}(L+g)}}{\left(3-d\right)e^{m_{c}(L+g)+m_{h}L}+\left(3+d\right)e^{m_{c}(L+g)-m_{h}L}-\left(3+d\right)e^{3m_{c}(L+g)+m_{h}L}-\left(3-d\right)e^{3m_{c}(L+g)-m_{h}L}}$$

with

(17) $$A=-\frac{J_{h}^{2}\rho_{0}}{k_{p}m_{h}^{2}}$$

(18) $$B=\frac{J_{c}^{2}\rho_{0}}{k_{p}m_{c}^{2}}-\frac{J_{h}^{2}\rho_{0}}{k_{p}m_{h}^{2}}$$

(19) $$D=-\frac{J_{c}^{2}\rho_{0}}{k_{p}m_{c}^{2}}$$

(20) $$F=-6A\left(\left(3+d\right)e^{m_{c}g}+\left(3-d\right)e^{-m_{h}L}\right)e^{m_{c}(L+g)}+3B\left(\left(3+d\right)e^{-2m_{h}L}+\left(3-d\right)e^{2m_{h}L}\right)e^{-m_{c}(L+g)}$$

(21) $$G=\left(3\cosh(m_{h}L)\sinh(m_{c}g)+d\sinh(m_{h}L)\cosh(m_{c}g)\right)\cosh(m_{c}L)$$

(22) $$H=\left(3\cosh(m_{h}L)\cosh(m_{c}g)+d\sinh(m_{h}L)\sinh(m_{c}g)\right)\sinh(m_{c}L)$$

For the deflection analysis of the actuators, the linear thermal expansion for both upper (*ΔL_h_*) and bottom (*ΔL_c_*) beams can be determined as:

(23) $$\Delta L_{h}=\alpha \int_{0}^{L}\left(T_{h}(s)-T_{0}\right)ds$$

(24) $$\Delta L_{c}=\alpha \int_{L}^{2L+g}\left(T_{c}(s)-T_{0}\right)ds$$

where *α* is the thermal expansion coefficient of polysilicon. 

By substituting Equations (8), (9) and (13)–(16) into Equations (23) and (24), the thermal expansions of the upper and bottom beams are given by:

(25) $$\Delta L_{h}=\alpha \left\{\frac{C_{1}}{m_{h}}\left(e^{m_{h}L}-1\right)-\frac{C_{2}}{m_{h}}\left(e^{-m_{h}L}-1\right)+\frac{J_{h}^{2}\rho_{0}L}{k_{p}m_{h}^{2}}\right\}$$

(26) $$\Delta L_{c}=\alpha \left\{\frac{C_{3}}{m_{c}}\left[e^{m_{c}L}\left(e^{m_{c}(L+g)}-1\right)\right]-\frac{C_{4}}{m_{c}}\left[e^{-m_{c}L}\left(e^{-m_{c}(L+g)}-1\right)\right]+\frac{J_{c}^{2}\rho_{0}(L+g)}{k_{p}m_{c}^{2}}\right\}$$

**Figure 6:**
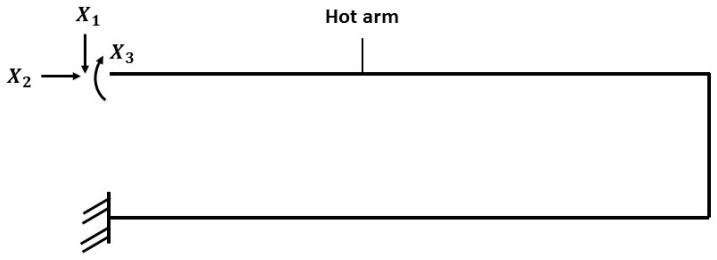
Rigid structure simplified for the electrothermal actuator regarding three redundant forces and moments (*X*_1_, *X*_2_ and *X*_3_).

The structure of the electrothermal actuator can be considered as a plane rigid frame with two fixed ends. This actuator (see Figure 6) has a statically indeterminate structure with the degree of indeterminacy of 3 [35,36]. The bending moment of the actuator structure due to three unknowns (*X*_1_, *X*_2_ and *X*_3_) is studied using the force method [35]. These unknowns are internal forces (horizontal force *X*_1_, vertical force *X*_2_ and bending moment *X*_3_). The force method will be used to find the redundant unknowns followed by the virtual work method to obtain the deflection at the tip of the frame.

The three redundants (*X*_1_, *X*_2_ and *X*_3_) are calculated through the canonical equations of the force method, which satisfy the compatibility conditions of the deformations [36]. For this case, the canonical equations are given by the following matrix form:

(27) $$\left[\begin{array}{ccc} \delta_{11} & \delta_{12} & \delta_{13}\\ \delta_{21} & \delta_{22} & \delta_{23}\\ \delta_{31} & \delta_{32} & \delta_{33} \end{array}\right]\left[\begin{array}{c} X_{1}\\ X_{2}\\ X_{3} \end{array}\right]=\left[\begin{array}{c} 0\\ \Delta L_{h}-\Delta L_{c}\\ 0 \end{array}\right]$$

where the coefficients $\delta_{ij}$ are called unit displacements that represent the displacements along the direction of unknown *X*_i_ caused by action of unit unknown *X*_j_. $\delta_{ij}$ can be determined by the diagram product of the bending moments related with the unit unknowns *X*_i_ and *X*_j_. These coefficients are obtained as:

(28) $$\delta_{11}=\frac{L^{2}}{3EI_{c}}\left(L+3g\right)+\frac{L^{3}}{3EI_{h}}$$

(29) $$\delta_{12}=\delta_{21}=-\frac{Lg}{2EI_{c}}\left(L+g\right)$$

(30) $$\delta_{13}=\delta_{31}=-\frac{L^{2}}{2EI_{h}}-\frac{L}{2EI_{c}}\left(L+2g\right)$$

(31) $$\delta_{22}=\frac{g^{2}}{3EI_{c}}\left(g+3L\right)$$

(32) $$\delta_{23}=\delta_{32}=\frac{g}{2EI_{c}}\left(g+2L\right)$$

(33) $$\delta_{33}=\frac{L}{EI_{h}}+\frac{1}{EI_{c}}\left(g+L\right)$$

where *E* is the Young’s modulus of polysilicon, *I_h_* and *I_c_* are the moment of inertia of the hot and cold beams, respectively. 

Taking at account the method of virtual work, a unit force *F* is applied to the free end of actuator to calculate the maximum out-of-plane displacement:

(34) $$\delta_{\max}=\int \frac{M_{F}M}{EI_{h}}ds=\frac{L^{2}}{6EI_{h}}\left(X_{1}L-3X_{3}\right)$$

where *M_F_* is the bending moment due to the virtual unit force and *M* the bending moment related with the thermal expansion. The physical and mechanical properties of the polysilicon used in the above analysis are listed in Table 2.

Solving Equation (27), the unknowns *X*_1_, *X*_2_ and *X*_3_ are the follows:

(35) $$X_{1}=\frac{18EI_{c}I_{h}\left(\Delta L_{h}-\Delta L_{c}\right)\left(I_{h}g+I_{c}L+I_{h}L\right)}{L\left(6I_{c}^{2}L^{3}+2I_{c}^{2}L^{2}g+6I_{c}I_{h}L^{3}+40I_{c}I_{h}L^{2}g+8I_{c}I_{h}Lg^{2}+2I_{h}^{2}L^{2}g+17I_{h}^{2}Lg^{2}+3I_{h}^{2}g^{3}\right)}$$

(36) $$X_{2}=\frac{6EI_{c}\left(\Delta L_{h}-\Delta L_{c}\right)\left(I_{c}^{2}L^{2}+2I_{c}I_{h}L^{2}+7I_{c}I_{h}Lg+I_{h}^{2}L^{2}+7I_{h}^{2}Lg+6I_{h}^{2}g^{2}\right)}{g^{2}\left[6I_{c}^{2}L^{3}+2I_{c}^{2}L^{2}g+6I_{c}I_{h}L^{3}+40I_{c}I_{h}L^{2}g+8I_{c}I_{h}Lg^{2}+2I_{h}^{2}L^{2}g+17I_{h}^{2}Lg^{2}+3I_{h}^{2}g^{3}\right]}$$

(37) $$X_{3}=\frac{6EI_{c}I_{h}L\left(\Delta L_{c}-\Delta L_{h}\right)\left(5I_{h}g-I_{c}g+I_{c}L+I_{h}L\right)}{g\left[6I_{c}^{2}L^{3}+2I_{c}^{2}L^{2}g+6I_{c}I_{h}L^{3}+40I_{c}I_{h}L^{2}g+8I_{c}I_{h}Lg^{2}+2I_{h}^{2}L^{2}g+17I_{h}^{2}Lg^{2}+3I_{h}^{2}g^{3}\right]}$$

Substituting Equations (35) and (36) into Equation (34), we determine the maximum out-of-plane displacement (*δ*_max_) of the electrothermal actuator:

(38) $$\delta_{\max}=\frac{3I_{c}L^{2}\left(\Delta L_{h}-\Delta L_{c}\right)\left(I_{c}L^{2}+I_{h}L^{2}+I_{h}g^{2}+6I_{h}Lg\right)}{g\left(6I_{c}^{2}L^{3}+2I_{c}^{2}L^{2}g+6I_{c}I_{h}L^{3}+40I_{c}I_{h}L^{2}g+8I_{c}I_{h}Lg^{2}+2I_{h}^{2}L^{2}g+17I_{h}^{2}Lg^{2}+3I_{h}^{2}g^{3}\right)}$$

## 3. Results and Discussions

This section presents the results of the temperature shift and displacements of the actuator caused by different bias voltages. For this, we considered several variations in the dimensions (width and length) of the actuator.

**Figure 7:**
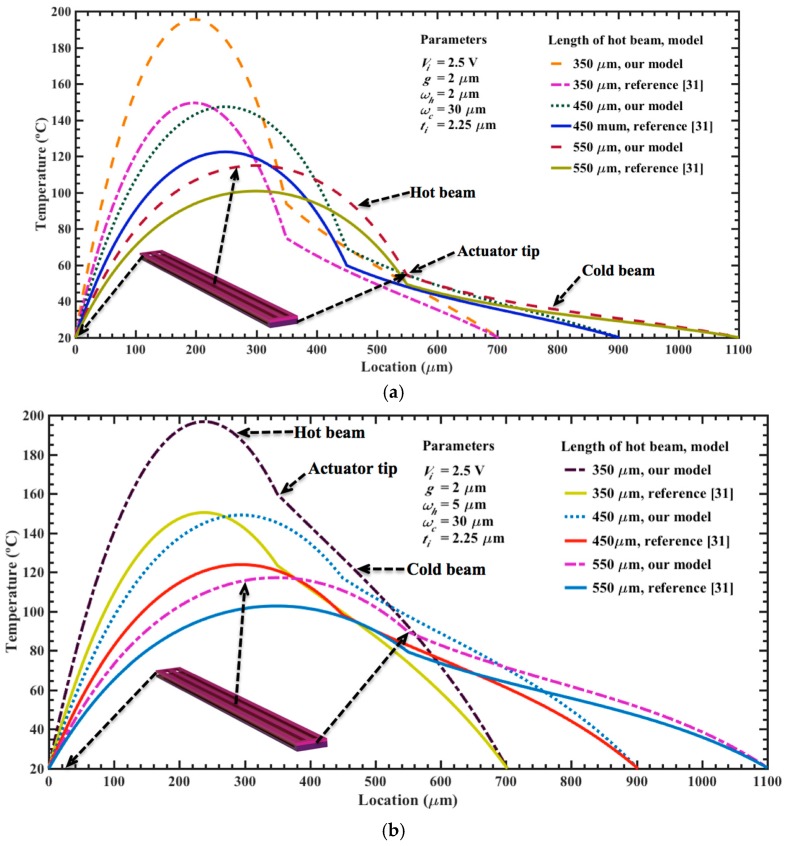
Distribution of the temperature along of the upper (hot) and bottom (cold) beams of an electrothermal actuator, which considers different lengths (350 μm to 550 μm) and two width values for the upper beams: (**a**) 2 μm; and (**b**) 5 μm.

**Figure 8:**
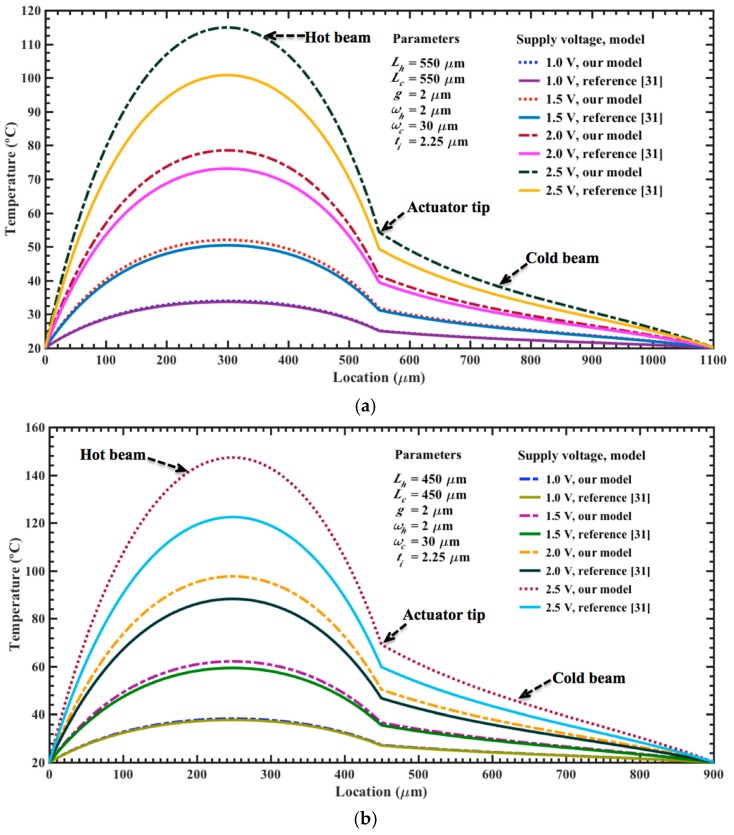
Distribution of the temperature along of the upper (hot) and bottom (cold) beams of two electrothermal actuators with lengths of (**a**) 550 μm and (**b**) 450 μm. This temperature is due to different bias voltages, whose values change from 0.5 to 2.5 V.

**Figure 9:**
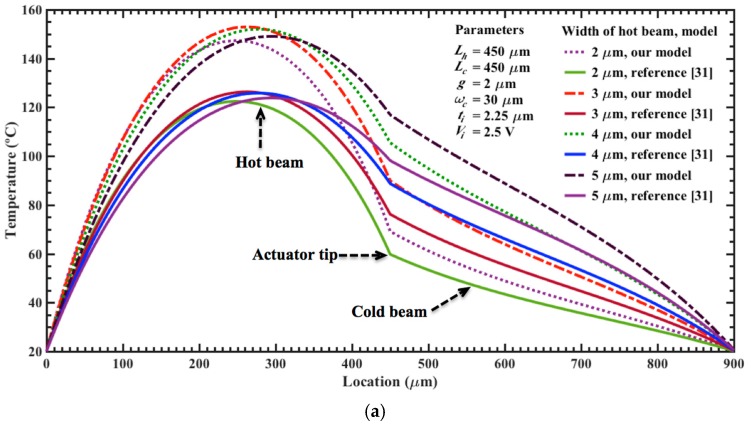

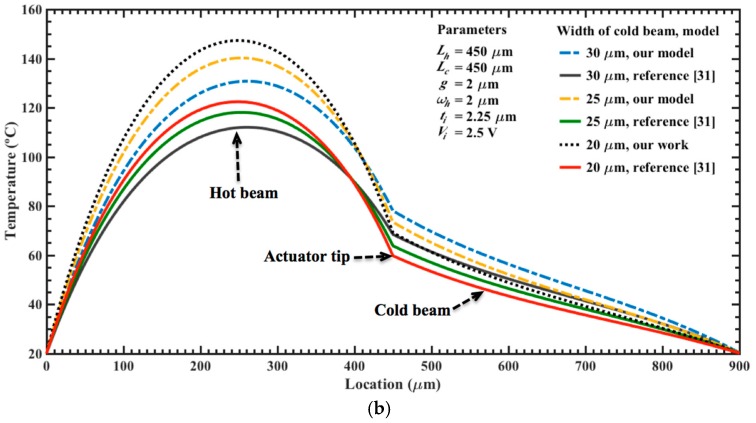
Distribution of the temperature along of the upper (hot) and bottom (cold) beams of an electrothermal actuator, modifying the width of the (**a**) upper and (**b**) bottom beams. For both cases, the length of the actuator is 450 μm and bias voltage is 2.5 V, respectively.

By using Equations (8), (9) and (38), we determine the temperature and maximum out-of-plane displacement of the electrothermal actuator generated by low dc bias voltages. In this analysis, the initial temperature of the actuator is 20 °C and the length of each actuator is modified between 350 and 550 μm. In addition, we regard a variable width (i.e., *w_h_* of 2 μm to 5 μm and *w_c_* of 20 μm to 30 μm) for the upper and bottom beams and a constant thickness (i.e., *t_h_* = *t_c_* = 2.25 μm). We compared the results of our models with respect to analytical models of temperature and displacements of electrothermal actuators reported by reference [31]. For this, we use Equations (7), (8) and (23) of reference [31] and assume negligible the flexure beam length (i.e., *L_f_* = 0). However, these models are applied for electrothermal actuators of variable cross-section area with in-plane deflections. In order to employ these models to our actuators with out-of-plane deflections, we considered that the variables of width and thickness of their hot and cold beams are equals to the thickness and width of our hot and cold beams. Figure 7a,b shows the results of the temperature along of the surface of the upper (hot) and bottom (cold) beams, which are generated by a bias voltage of 2.5 V. This distribution considers different lengths (350 and 550 μm) and two values of width for each upper beam (2 and 5 μm). For all the cases, the maximum temperature is achieved close to the half of the length of the upper beam. The shorter beams present higher temperatures than the larger beams due to their less electrical resistance, which produce higher currents for a bias voltage. For the upper beams of 5 μm width, the temperature decays more slowly along of the electrothermal actuator, as shown in Figure 7b. In the actuator tip, we observed a significant variation in the behavior of the temperature distribution along of the hot and cold beams. The results of our analytical models have a similar behavior respect to those of reference [31]; although, our results register the highest temperature values in all the cases. Next, we calculate the temperature distribution regarding two actuators of different lengths (450 and 550 μm), which are supplied by different dc bias voltages, as shown in Figure 8a,b. The maximum voltage of 2.5 V generates the higher temperature magnitudes (147.3 °C and 114.9 °C) for both actuators, considering our models. For the actuator of 450 μm length, the bias voltages of 1.0 V, 1.5 V and 2.0 V increase the temperature up 38.2 °C, 62.1 °C and 97.6 °C, respectively. For the same voltages, the actuator of 550 μm length has an increment of temperature of 34.0 °C, 52.0 °C and 78.5 °C, respectively. Also, the temperature distribution along the actuator of 450 μm length was determined varying the width of the upper and bottom beams, as shown in Figure 9a,b. For upper beams of 2 μm width and bias voltage of 2.5 V, the temperature has a low increment of 16.6 °C when the width of the bottom beam increases from 20 to 30 μm. Instead, the temperature distribution decays more slowly for upper beams of 5 μm width, keeping 30 μm width for the bottom beam. For these cases, the results of our models have good agreement respect to those of reference [31].

**Figure 10:**
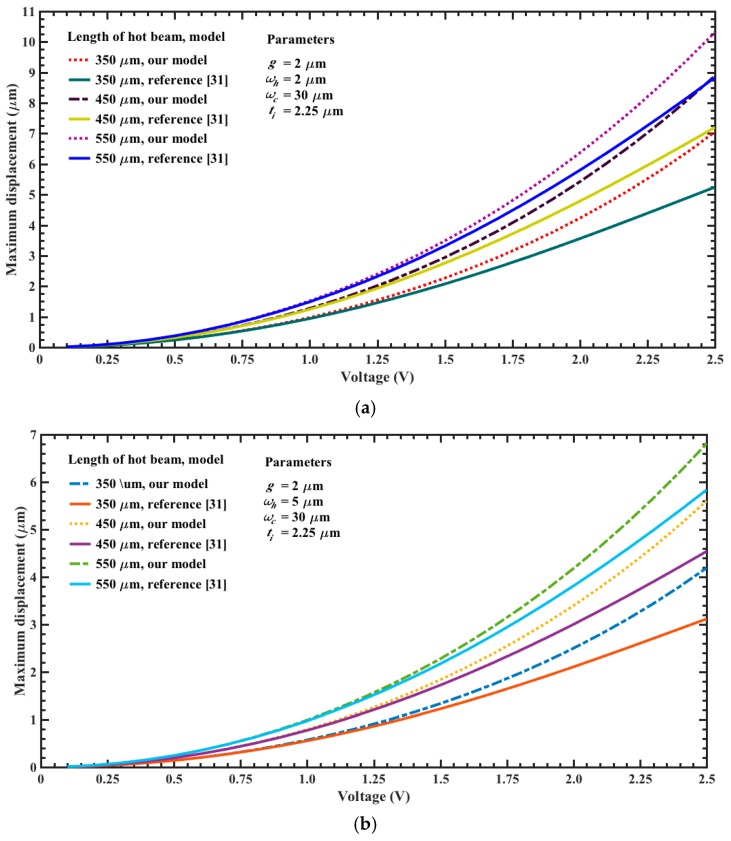
Maximum out-of-plane displacements of the electrothermal actuator tip as a function of bias voltage, regarding different lengths and two width values for the upper beams: (**a**) 2 μm and (**b**) 5 μm.

**Figure 11:**
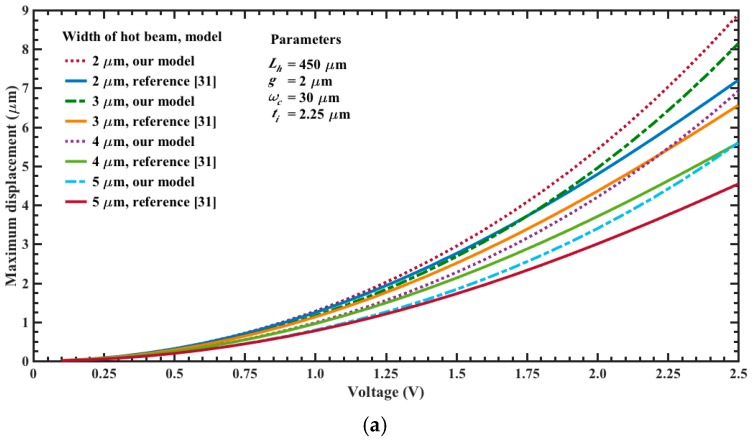

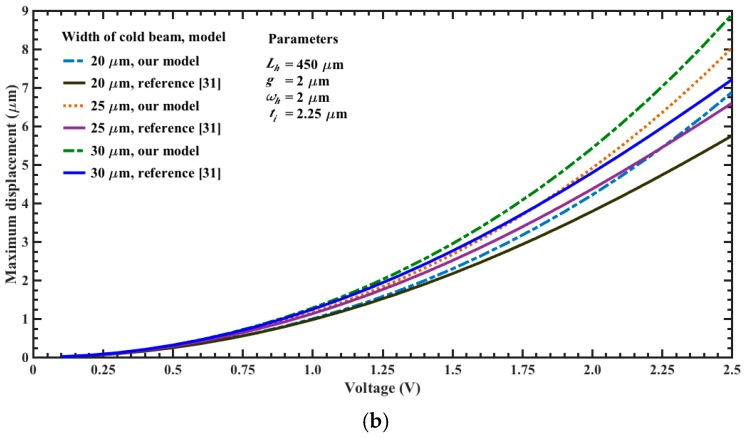
Maximum out-of-plane displacements of the electrothermal actuator tip as a function of bias voltage, varying the width of the (**a**) upper and (**b**) bottom beams. For both cases, the length of the actuator is 450 μm and bias voltage is 2.5 V, respectively.

Figure 10a,b depicts the maximum out-of-plane displacements of the actuator tip as a function of bias voltage and assuming different length and width values. For these cases and considering 2.5 V, the beam of 550 μm length has the larger displacements (10.3 μm and 6.8 μm) when *w_h_* = 2 μm and *w_c_* = 30 μm, respectively. These displacements have direction down due to the higher temperature of the upper beams. However, if the position of the beams is inverted then the motion of the actuator will be upward. If the length of the actuator is 450 μm and the bias voltage is 2.5 V then the maximum displacements are 8.9 μm and 5.6 μm, respectively. The response of our models has good agreement respect to results of reference [31]. Although the displacements obtained with our analytical models have higher values than those of the reference [31]. Figure 11a,b shows the maximum out-of-plane displacements of the actuator (450 μm length and 2.5 V voltage) considering different dimensions in the width of its upper and bottom beams. For these cases, the larger displacement (8.9 μm) is obtained with 2.5 V voltage for beams with *w_h_* = 2 μm and *w_c_* = 30 μm, respectively. In addition, the displacement of the actuator tip decreases when the width of the upper beams increases. Moreover, if the width of the bottom beam increases then the actuator tip will have larger displacements. The electrical power of each actuator is determined using the equivalent electrical circuit of Figure 5. For an actuator with *w_h_* = 2 μm, *w_c_* = 30 μm and three different lengths *L_h_*: 350 μm, 450 μm and 550, we obtain the following electrical power: 9.9 mW, 7.7 mW and 6.3 mW. Finally, the displacements of the actuator tip can be increased with bias voltages higher than 2.5 V, which also will increment the electrical power. For instance, if the actuator of *L_h_* = 550 μm and *w_h_* = 2 μm is biased with 5 V then its maximum displacement, temperature and power are increased up 59.2 μm, 570.3 °C and 25.2 mW, respectively. Furthermore, the mirror surface area can be scalable to achieve larger values than 10000 μm^2^. On the other hand, the surface of the silicon substrate below of the actuators array and mirror must be etched using DRIE process to allow the free motion of the actuators and mirror under different bias voltages. Nevertheless, the maximum displacement of the actuators must generate stress less than the rupture stress of the polysilicon. 

**Figure 12:**
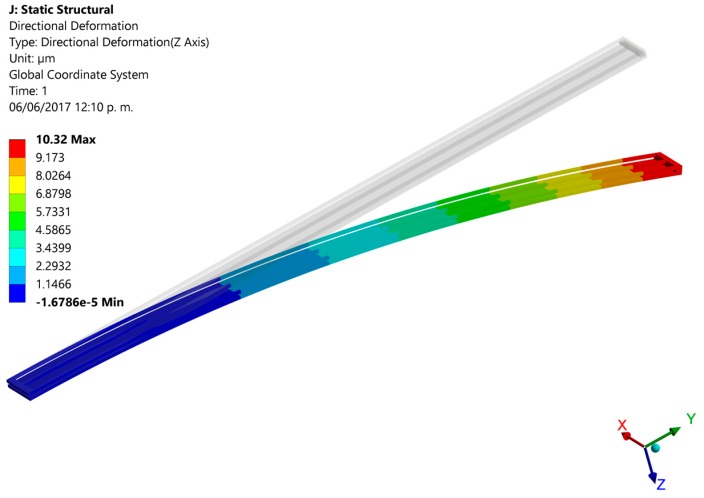
Out-of-plane displacements of one polysilicon electrothermal actuator (*L_h_* = *L_c_* = 550 μm) caused by a 2.5 V bias voltage.

**Figure 13:**
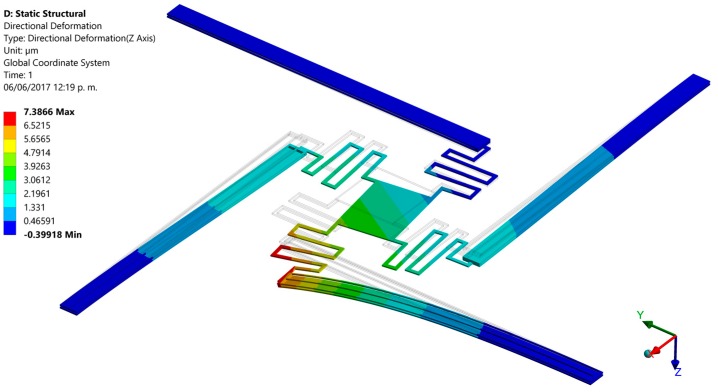
Out-of-plane deflections of the MEMS mirror when one polysilicon electrothermal actuator (*L_h_* = *L_c_* = 550 μm) is biased with 2.5 V.

**Figure 14:**
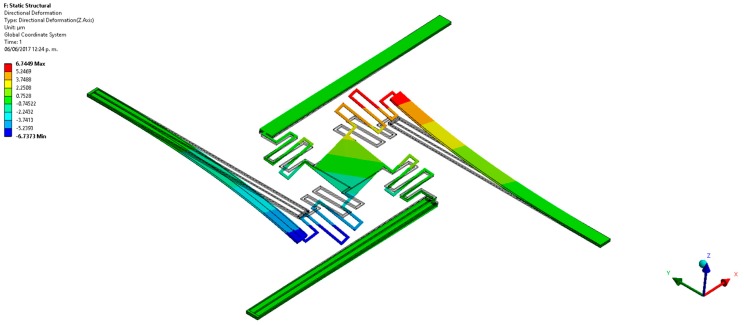
Out-of-plane displacements of the MEMS mirror when two polysilicon electrothermal actuators (*L_h_* = *L_c_* = 550 μm) are biased with 2.5 V.

**Figure 15:**
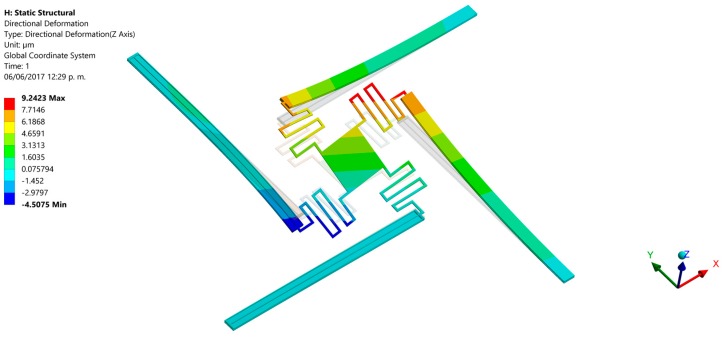
Out-of-plane displacements of the MEMS mirror when three polysilicon electrothermal actuators (*L_h_* = *L_c_* = 550 μm) are biased with 2.5 V.

**Figure 16:**
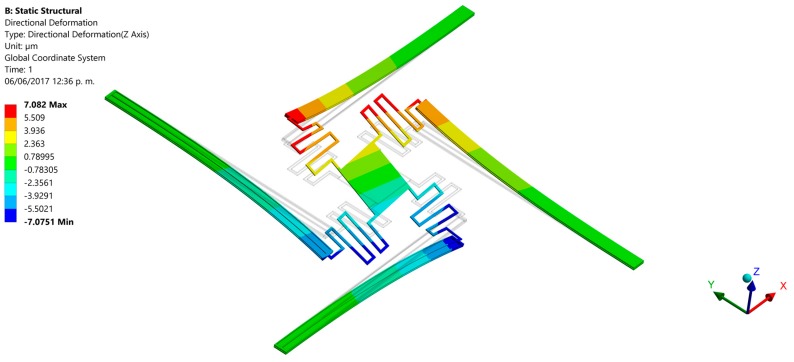
Out-of-plane displacements of the MEMS mirror when four polysilicon electrothermal actuators (*L_h_* = *L_c_* = 550 μm) are biased with 2.5 V.

Finally, we developed finite elements method (FEM) models using the ANSYS^®^ software (version 15.0, ANSYS, Berkeley, CA, USA) to predict the out-of-plane displacements of the proposed actuation mechanism. For this, the pads were negligible and the initial end of each actuator was considered as fixed support. For these supports were applied a bias voltage of 2.5 V and initial temperature of 20 °C. The FEM models regard polysilicon actuators with the following dimensions: *L_h_* = *L_c_* = 550 μm, *ω_h_* = 2 μm, *ω_c_* = 30 μm, *t_i_* = 2.25 μm and *g* = 2 μm. Our FEM models include elements solid226 type with a hexahedral mesh. First, we use a FEM model of a single electrothermal actuator under 2.5 V bias voltage. Figure 12 depicts the out-of-plane displacements of this actuator, achieving a maximum downward deflection of 10.3 μm that well agree with the results (10.3 μm and 8.8 μm) of both our analytical model and that of the reference [31], as shown in Figure 10a. Next, we used a FEM model composed by four polysilicon electrothermal actuators, four springs (508 μm length, 5 μm width and 2.25 μm thickness) and a mirror. Each one of these actuators has the same dimension respect to the previous actuator. The initial ends of the four actuators have boundary conditions of clamped support and temperature of 20 °C. For this FEM model, we studied four different cases modifying the bias voltage values of the four actuators. For the first case, one actuator was only supplied with a voltage of 2.5 V, keeping the other three actuators without bias voltage (see Figure 13). Thus, the actuator and mirror have maximum out-of-plane deflections of 7.4 μm and 4.8 μm, respectively. For this case, the displacement of the actuator decreases (3.9 μm) respect the response of a single actuator without connection with springs and mirror. This displacement reduction is due to an increment of the model stiffness when the four actuators are joined to the mirror. In the second case two actuators are biased with 2.5 V, obtaining out-of-plane displacements with opposite directions (downward and upward) that allow the mirror rotation with respect to two of its vertices, as shown in Figure 14. The absolute value of the maximum displacement of the two biased actuators is 6.7 μm, which is 3.5 μm less than that obtained with a single actuator. Two mirror vertices reach maximum displacements of 3.7 μm and −3.7 μm, respectively. For the third case, a 2.5 V bias voltage is applied for three actuators, achieving maximum displacements of 9.2 μm, 7.7 μm and −4.5 μm (see Figure 15). Indeed, two mirror vertices have displacements of 6.2 μm and −1.4 μm that enable the mirror tilting. In the last case all the actuators are biased with 2.5 V, obtaining the downward and upward deflection of two actuator pairs as well as the mirror rotation along the *x*-axis (see Figure 16). The larger displacements of the actuators and mirror are 7.1 μm, −7.1 μm, 3.9 and −3.9 μm, respectively. In order to reach larger deflection and tilting of the actuators and mirror, the bias voltage can be increased. Moreover, the rotation orientation of the mirror can be regulated through the selective biasing of the four actuators. Also, the proposed actuation mechanism can be employed for MEMS mirrors of larger surface area and their rotation angles can be controlled using different bias voltages. 

Table 3 depicts the characteristics of several MEMS mirrors that use electrothermal actuators. Based on these devices, our design provides an easy actuation mechanism that does not require materials layers with different thermal expansion coefficients. It can simplify the actuators fabrication process and reduce the thermal residual stresses due to the fabrication. The proposed design is based on SUMMiT V process, which improves the flatness of the structures and minimize thermal residual strains. Indeed, our design has a minimum footprint size (1028 × 1028) and mirror surface area (100 μm × 100 μm), achieving different rotation orientations of the mirror that are well controlled using reduced bias voltages. Most of the other designs need different metallic films (e.g., Al, Cu, W or Pt) deposited on the actuators by sputtering process, which can generate initial thermal strains (i.e., initial displacement offset) that can affect the actuators performance. Indeed, our actuation mechanism can be adjusted for MEMS mirrors with larger surface area than 10,000 μm^2^, which can be suitable for potential applications in endoscopic OCT systems. 

## 4. Conclusions

The design and modeling of an electrothermal actuation mechanism for a polysilicon mirror (100 μm × 100 μm × 2.25 μm) was developed. These actuators were designed based on the SUMMiT V surface micromachining process from Sandia National Laboratories. The actuators are composed by two polysilicon structural layers, which are vertically separated by 2 μm. The temperature and out-of-plane displacements of the actuators were determined using electrothermal and structural models and assuming the polysilicon resistivity as a function of temperature. The electrothermal models included the rate of heat energy generation, heat conduction and heat energy loss. On the other hand, the structural model was obtained with the force method and assuming low dc voltages (0.5 V to 2.5 V). For actuators with lengths of 450 and 550 μm, the higher temperatures and out-of-plane displacements generated by 2.5 V are: 147.3 °C, 115 °C, 8.9 μm and 10.3 μm, respectively. These actuators can have upward and downward motion if their structural layers are inverted. Thus, the mirror tilting can be controlled modifying the position of the structural layers and altering the actuators dimensions and magnitudes of the dc bias voltages. In addition, the device footprint size is 1028 μm × 1028 μm considering electrothermal actuators of 550 μm length. With a bias voltage of 2.5 V, the electrical power for an actuator of 550 μm length was 6.3 mW. The proposed actuation mechanism could be used to obtain the rotation of MEMS mirrors with different surface area. The rotation orientation of the mirrors can be modified through the selective biasing of the actuators. This actuation mechanism for MEMS mirrors could be considered for potential applications in endoscopic OCT systems.

Future researches will include the fabrication and characterization of several electrothermal actuators array for MEMS mirrors with different surface area using the SUMMiT V process. 

## Figures and Tables

**Table 1:** Resistance values of the equivalent electrical circuit of an electrothermal actuator considering the following dimensions: *ω_h_* = 2 μm, *ω_c_* = 30 μm and *t_h_* = *t_c_* = 2.25 μm.

Parameter	Electrical Resistance (Ω)		
	*L_h_* = 350 μm	*L_h_* = 450 μm	*L_h_* = 550 μm
*R*_1_	1576.6	2027	2077.4
*R*_2_	105.1	135.1	165.2
*R*_3_	1.7	1.7	1.7

**Table 2:** Physical and mechanical properties of the polysilicon beams.

Property	Value
Young’s Modulus, E	169 GPa
Thermal expansion, α	2.5 × 10^−6^ K^−1^
Thermal conductivity, $k_{p}$	125 W·m^−1^·K^−1^
Substrate Temperature, $T_{0}$	300 K
Linear temperature coefficient, ξ	1.25 × 10^−3^ K^−1^
Resistivity at $T_{0}$, $\rho_{0}$	20.27 × 10^−6^ Ω·m
Density	2330 kg·m^−3^
Poisson ratio	0.23

**Table 3:** Characteristics of several MEMS mirrors based on electrothermal actuators.

Authors	Mirror Size	Device Footprint (μm × μm)	Maximum Displacement (μm)	Bias Voltage (V)
Zhang et al. [18]	900 μm × 900 μm	2500 × 2500	312	3
Kawai et al. [37]	3000 μm diameter	5000 × 5000	*-	20
Zhang et al. [38]	1000 μm × 1000 μm	1500 × 1500	70	2
Li et al. [39]	1000 μm diameter	2000 × 2000	227	0.8
Espinosa et al. [40]	1000 μm × 1000 μm	1500 × 1500	174	3.5
Koh et al. [41]	1500 μm × 1000 μm	6000 × 6000	*-	5
Our work	100 μm × 100 μm	1028 × 1028	59.2	5

*- Data not available in literature.
